# Alkaline Sodium Hypochlorite Irrigant and Its Chemical Interactions

**DOI:** 10.3390/ma10101147

**Published:** 2017-09-29

**Authors:** Patricia P. Wright, Bill Kahler, Laurence J. Walsh

**Affiliations:** The University of Queensland School of Dentistry, Brisbane 4006, Australia; wyattkahler@bigpond.com (B.K.); l.walsh@uq.edu.au (L.J.W.)

**Keywords:** endodontic irrigant interactions, clinical implications, sodium hypochlorite, EDTA, citric acid, etidronate, chlorhexidine, MTAD, alexidine, octenisept

## Abstract

Endodontic irrigating solutions may interact chemically with one another. This is important, because even when solutions are not admixed, they will come into contact with one another during an alternating irrigation technique, forming unwanted by-products, which may be toxic or irritant. Mixing or alternating irrigants can also reduce their ability to clean and disinfect the root canal system of teeth by changing their chemical structure with subsequent loss of the active agent, or by inducing precipitate formation in the root canal system. Precipitates occlude dental tubules, resulting in less penetration of antimicrobials and a loss of disinfection efficacy. Sodium hypochlorite is not only a very reactive oxidizing agent, but is also the most commonly used endodontic irrigant. As such, many interactions occurring between it and other irrigants, chelators and other antimicrobials, may occur. Of particular interest is the interaction between sodium hypochlorite and the chelators EDTA, citric acid and etidronate and between sodium hypochlorite and the antimicrobials chlorhexidine, alexidine, MTAD and octenisept.

## 1. Introduction

The use of sodium hypochlorite (NaOCl) dates back over 100 years, when Dakin, recognizing its great reactivity and its ability to chemically digest organic matter, reported the use of an 0.5% solution for wound disinfection [1]. Its application to root canal therapy is credited to Alfred Walker in 1936 [2]. The unique ability of NaOCl to both dissolve organic tissue and to disinfect has made it the irrigant of choice in contemporary endodontics [3,4]. NaOCl will not, however, remove smear layer, so it is used in conjunction with a chelating agent to remove inorganic components [5]. Ethylenediaminetetraacetic acid (EDTA) is utilized mostly for this purpose [6]. However, chelators such as citric acid or other organic acids have been used [5]. More recently, the bisphosphonate etidronate has been assessed for mixing with NaOCl [7]. 

When tested on mature biofilms of clinical isolates of endodontic infection, NaOCl has superior antimicrobial activity compared to several other irrigants; however, it neither totally eradicates biofilm volume, nor achieves complete killing of bacteria [8]. Strategies to augment its use for disinfection include combination with other antimicrobial agents [9] or chelators [7] in the same solution, the use of a rinse sequence employing NaOCl with EDTA or other chelator or antimicrobial agent [10], and flushing the root canal with a solution possessing additional disinfection ability following irrigation with NaOCl [11]. 

NaOCl solutions used in endodontic irrigation range in concentration from 0.5–6% [6]. The pH and hydroxide ion (OH^−^) concentration vary significantly according to brand, and can range between 10.9 to 12.0 for pH, and from 0.03–1.1% for OH^−^ concentration [12]. There are products available with more OH^−^ that have a higher pH. It is also worth noting that endodontics is often performed with commercial bleaches used for general cleaning, such as Chlorox, which may result in the use of inappropriately high levels of NaOCl [13]. The reactions of NaOCl with other irrigants, as well as its clinical properties are better appreciated by understanding the various chemical species present at different pH levels. At pH values of 9 and above, the hypochlorite ion (OCl^−^) predominates. At neutral pH, hypochlorous acid (HOCl) is mostly present, while below pH 4, chlorine gas starts to form [14]. The antibacterial ability is maximized at pH conditions where HOCl predominates. In contrast, at higher pH levels where OCl^−^ is present, tissue dissolution is maximized [15]. The pH of NaOCl solutions can be lowered by its reaction with other irrigants [16] or when it comes into contact with dentine [17], and thus, there is potential for hypochlorite to be present in multiple forms. 

NaOCl is a strong oxidizing agent with a reduction potential of 1.6 *E°/V* [18]. The oxidation state of the chlorine atom in NaOCl is +1. Chlorine then acts as an electrophile, reacting with electron sources such as aromatic rings, doubly bonded carbon atoms, amino and sulphydryl groups [14]. The reaction with amino groups results in chloramine formation [14]. Additionally, OH^−^ is a nucleophile, and can drive chemical reactions [19]. 

## 2. Reactions of Sodium Hypochlorite with Chelating Agents 

### 2.1. Reactions of Sodium Hypochlorite with Ethylenediaminetetraacetic Acid

#### 2.1.1. Chemistry 

EDTA is used commonly at concentrations of 15% or 17%. Aqueous solutions are prepared by dissolving the di- or trisodium salts, resulting in a neutral to slightly alkaline pH [3,20]. At this pH, the reaction between NaOCl and EDTA is exothermic [21], and mixing NaOCl with EDTA results in a rapid and dramatic decrease of free available chlorine [7,21,22]. Gas formation has been observed from this combination [23], and low level chlorine gas emissions have been measured [20]. This interaction has been attributed to an acid/base neutralization reaction between NaOCl and EDTA, with the formation of HOCl, which then subsequently decomposes, releasing chlorine and oxygen, according to Equation (1) below [20].

4 HOCl **⇌** 2 Cl_2_ + O_2_ + 2 H_2_O (1)

The remaining free available chlorine in a 1:1 mixture of 1% NaOCl and 17% EDTA at 1 min is 10%, but this declines to 0% at one hour after mixing [7]. The decomposition of HOCl occurs when the pH is less than 4. This is much lower than the pH of the standard EDTA/NaOCl combination, which is 7.9–8.0 up to one hour from mixing [16]. However, the presence of chloride ions can shift the equilibrium, according to Le Chatelier’s principle, to favor chlorine formation, as in Equation (2). Some formulations of NaOCl are known to have high sodium chloride levels [21], thus encouraging this effect [23].

HOCl + Cl^−^ + H^+^ ⇌ Cl_2_ + H_2_O (2)

In the above reactions, the EDTA molecule is left intact [20]. However, as nuclear magnetic resonance ^1^H spectroscopy (^1^H NMR) studies have identified unspecified and slowly formed EDTA breakdown products [24], these are not the only reaction mechanisms in operation. The formation of chloramines has been suggested [21], which would occur via chlorine electrophilic attack on EDTA amino groups. 

Recently, more alkaline solutions have been achieved by mixing the tetrasodium salt of EDTA (Na_4_EDTA) with NaOCl. This combination results in the longer maintenance of both free available chlorine and desirable pH conditions [16], although concentrations of both EDTA and NaOCl need to be reduced to obtain this result [25]. The pH of a 1:1 mixture of 5% NaOCl and 10% Na_4_EDTA falls from 11.9 at 10 min after mixing to 9.0 at one hour after mixing, during which period the remaining free available chlorine declines from 90% to 62% [16]. 

#### 2.1.2. Clinical Implications 

For mixtures of EDTA and NaOCl that have a low pH, the loss of free available chlorine significantly reduces the ability of NaOCl to dissolve organic tissue [22,26]. Thus, these irrigants should neither be mixed, nor come into contact with one another. The chelating ability of EDTA [7] and the disinfecting capacity of NaOCl are, however, maintained [7,22]. 

This is in contrast to more alkaline mixtures using Na_4_EDTA, which, when combined with NaOCl, maintain some ability to dissolve organic matter. When 5% NaOCl is mixed 1:1 with 10% Na_4_EDTA, resulting in 2.5% NaOCl and 5% Na_4_EDTA, tissue dissolution at 15 min is comparable to that of 2.5% NaOCl, resulting in a short therapeutic window [16]. 

Also of clinical relevance, a case report attributing a subcutaneous emphysema to gas formation, resulting from the interaction of RC-Prep with NaOCl, has been reported [27]. RC-Prep contains both EDTA and urea peroxide and reactions of these components with NaOCl is possible.

### 2.2. Reactions of Sodium Hypochlorite and Citric Acid

#### 2.2.1. Chemistry 

Citric acid is a tricarboxylic acid and a tridentate ligand that will chelate Ca^2+^. When NaOCl and citric acid are mixed, the resulting pH is considerably lower than the mixture of NaOCl and EDTA. A 1:1 mixture of 2.5% NaOCl and 10% citric has a pH of 3.1 [28]. Low pH levels favors chlorine formation, and production of this gas has been measured in a 1:1 mixture of 5.25% NaOCl and 50% citric acid at 7 times the level of that of in a 1:1 mixture of 5.25% NaOCl and 15% EDTA [20]. However, bubble formation in mixtures of NaOCl and citric acid has been observed at the much lower citric acid concentration of 10% when combined with 5.25%, 2.5% and 1% solutions of NaOCl [23]. Free available chlorine is depleted rapidly from combinations of NaOCl and citric acid [7,28], with a 1:1 mixture of 1% NaOCl: 10% citric acid losing all of its free available chlorine after only one minute [7].

#### 2.2.2. Clinical Implications 

As can be expected from the observations of chlorine formation and loss of free available chlorine, mixtures of NaOCl and citric acid lose their ability to dissolve organic tissue [10]. Citric acid and NaOCl should then not be present together during root canal therapy, either admixed or used sequentially. However, citric acid in combination with NaOCl does maintain both its chelation [7] and antimicrobial action [7,28]. 

### 2.3. Reactions of Sodium Hypochlorite with Etidronate

Because of the above interactions of NaOCl with EDTA and citric acid, alternative chelators to use in mixtures with NaOCl have been sought. Etidronate has emerged as one such possible chelator, and has been tested in these mixtures at concentrations of 9% and 18% [25,29]. 

#### 2.3.1. Chemistry 

Etidronate is a chelator of calcium ions [7]. Little has been documented of the chemical reaction between NaOCl and etidronate; however, it is thought to break down to acetic acid [25]. The pH range of a solution containing 5% NaOCl and 18% etidronate up to one hour after mixing is 8.6–8.7 [25]. The pH range is, however, dependent on the NaOCl concentration, with lower concentrations having a higher pH [25]. It can thus be expected that over a 60-min period, as a basic solution is maintained, NaOCl is predominantly in the OCl^−^ form. Free available chlorine is lost at a considerably slower rate when NaOCl is combined with etidronate compared to when it is mixed with di- or trisodium EDTA or citric acid. A 1:1 mixture of 1% NaOCl: 9% etidronate maintains all of its free available chlorine at 1 min, and this declines to 80% at 60 min and to 20% after 24 h [7]. This mixture loses less free available chlorine compared to the mixture of NaOCl with Na_4_EDTA [25].

#### 2.3.2. Clinical Implications 

Due to the maintenance of higher pH and free available chlorine levels in the mixture of NaOCl with etidronate, a therapeutic window of one hour exists, with acceptable levels of organic tissue dissolution being recorded within this timeframe [29].

Comparing the use of an etidronate/NaOCl combination to a final rinse of neutral pH EDTA, the calcium chelation of the former is half to two thirds less [7,30], although it appears sufficient to remove smear layer [30]. The ability of NaOCl to disinfect the root canal is maintained in the combination [31]. 

## 3. Reactions of NaOCl with Antimicrobial Agents or Antimicrobial/Chelator Mixtures 

### 3.1. Reactions of Sodium Hypochlorite with Chlorhexidine

#### 3.1.1. Chemistry 

Chlorhexidine (CHX) is a cationic bisbiguanide with broad spectrum antimicrobial actions, particularly against Gram-positive bacteria [32]. The two biguanide units are linked by an aliphatic chain, and both are terminated by 4-chlorophenyl side groups [33]. When solutions of CHX and NaOCl come into contact, an orange-brown precipitate is formed [34,35]. There has been considerable debate about the composition of the precipitate, with different analytical techniques showing contradictory results. The presence of parachloroanaline (PCA) was reported in studies based on time-of-flight ion mass spectroscopy [34,36], gas chromatography/mass spectroscopy (GC/MS) [37] or combined ^1^H NMR/chemical analysis [35], whilst in other studies using ^1^H NMR [19,38], no PCA was detected. Finally, a decisive study employing a variety of techniques including high performance, thin layer and gas chromatography techniques, ^1^H NMR, infrared spectroscopy and GC/MS showed that PCA was not present in the precipitate [39]. Chlorophenylurea has been proposed as one of the components of the precipitate [19].

#### 3.1.2. Clinical Implications 

CHX has been suggested for use in endodontic irrigation as a final rinse following NaOCl irrigation to achieve more bacterial killing than NaOCl alone [6]. This is particularly the case for retreatments when *Enterococcus faecalis* may be present [40]. The precipitation products, although not positively identified, are worrying because of their probable similarity to the known toxin, chloroguanidine [19]. Additionally, these precipitates have been found within dentinal tubules [36] and in a layer on the main canal where they occlude the canal orifice [41,42], reducing dentine permeability and lowering the efficacy of endodontic irrigants [43]. Lastly, any colored material in the root canal has the potential to stain dentine. 

### 3.2. Reactions of Sodium Hypochlorite with Alexidine

#### 3.2.1. Chemistry 

Alexidine (ALX) is a bisbiguanide that is similar in structure to CHX, except that the side chains consist of 2-ethylhexyl groups instead of the chlorophenyl groups of CHX [33]. In its reaction with NaOCl, having no aromatic components, the question of PCA by-product formation is non-existent, and this has been shown to be the case [44]. When 4% NaOCl is added to ALX, a pale-yellow cloudy reaction product is observed with ALX concentrations of 1%, 0.5%, 0.25%, fading to clear for a 0.125% solution [44]. 

#### 3.2.2. Clinical Implications

The yellow reaction by-product has not been identified, and thus its toxicity is yet to be determined. Compared to the blocked dentinal tubules observed from the CHX/NaOCl combination, scanning electron microscopy (SEM) shows root canal irrigation with an ALX/NaOCl mixture results in largely patent dentinal tubules, although it is unclear how this situation compares to alternating NaOCl and EDTA solutions [44].

### 3.3. Reactions of Sodium Hypochlorite with MTAD

#### 3.3.1. Chemistry 

MTAD is an endodontic irrigant containing 3% doxycycline, 4.25% citric acid and 0.5% polysorbate 80 [11,45]. The latter is a detergent. Doxycycline and other tetracyclines are not only antibacterial, but also are calcium chelators [46]. NaOCl is thought to oxidize the doxycycline component of MTAD when these irrigants are mixed [47]. In benchtop experiments, the reaction of the two results in the immediate formation of a yellow-green precipitate, which develops into a purple-brown color with time and light exposure [47]. Adding the reducing agent ascorbic acid to NaOCl before mixing with MTAD can stop precipitate formation [47].

#### 3.3.2. Clinical Implications 

Because of their calcium chelation ability, tetracyclines have a high affinity for tooth structure [46], and dark brown staining can occur when root canals are irrigated sequentially by NaOCl and MTAD [47]. This irrigation sequence also results in a reduction of the substantivity of the MTAD antimicrobial action. Clinically, this can be avoided by neutralizing the oxidizing action of NaOCl with a rinse of ascorbic acid before the MTAD is applied into the root canal [47]. 

### 3.4. Reactions of Sodium Hypochlorite with Octenisept

#### 3.4.1. Chemistry 

Octenisept^TM^ is an antimicrobial containing 0.1% octenidine and 2% phenoxyethanol [48]. Octenidine is a dicationic bispyridinamine compound [38,49] with antimicrobial activity against endodontic pathogens [50]. The cloudy white substance that forms when NaOCl is added to octenisept has been identified as phenoxyethanol, which precipitates out of solution upon mixing [48]. Mixtures of NaOCl and octenisept do not lose free available chlorine over time [9].

#### 3.4.2. Clinical Implications 

Recent research on the NaOCl/octenisept combination has highlighted that there are few clinical implications, because of the lack of interactions between these. Moreover, phenoxyethanol precipitates only minimally occlude dentinal tubules [9,48]. Further research into the NaOCl/octenisept combination is needed in order to establish if this new mixture has advantages over existing protocols. 

## 4. Conclusions

The mixing of sodium hypochlorite with other endodontic irrigants can result in not only a lowering of the pH of the hypochlorite component and its decomposition to chlorine gas, but also in the formation of unwanted by-products originating from the added irrigant. These two processes have implications in terms of clinical performance and toxicity. Rapid loss of free available chlorine inactivates the tissue dissolution capacity of sodium hypochlorite and precipitate formation can occlude dentinal tubules. Complete chemical analysis of breakdown products remains an important objective whenever considering combining agents with sodium hypochlorite. 
